# Radiomics of Musculoskeletal Sarcomas: A Narrative Review

**DOI:** 10.3390/jimaging8020045

**Published:** 2022-02-13

**Authors:** Cristiana Fanciullo, Salvatore Gitto, Eleonora Carlicchi, Domenico Albano, Carmelo Messina, Luca Maria Sconfienza

**Affiliations:** 1 Scuola di Specializzazione in Radiodiagnostica, Università degli Studi di Milano, 20122 Milan, Italy; eleonora.carlicchi@unimi.it; 2 Dipartimento di Scienze Biomediche per la Salute, Università degli Studi di Milano, via Riccardo Galeazzi 4, 20161 Milan, Italy; luca.sconfienza@unimi.it; 3 IRCCS Istituto Ortopedico Galeazzi, 20161 Milan, Italy; albanodomenico@me.com (D.A.); carmelomessina.md@gmail.com (C.M.); 4 Sezione di Scienze Radiologiche, Dipartimento di Biomedicina, Neuroscienze e Diagnostica Avanzata, Università degli Studi di Palermo, 90127 Palermo, Italy

**Keywords:** artificial intelligence, musculoskeletal, radiomics, sarcoma

## Abstract

Bone and soft-tissue primary malignant tumors or sarcomas are a large, diverse group of mesenchymal-derived malignancies. They represent a model for intra- and intertumoral heterogeneities, making them particularly suitable for radiomics analyses. Radiomic features offer information on cancer phenotype as well as the tumor microenvironment which, combined with other pertinent data such as genomics and proteomics and correlated with outcomes data, can produce accurate, robust, evidence-based, clinical-decision support systems. Our purpose in this narrative review is to offer an overview of radiomics studies dealing with Magnetic Resonance Imaging (MRI)-based radiomics models of bone and soft-tissue sarcomas that could help distinguish different histotypes, low-grade from high-grade sarcomas, predict response to multimodality therapy, and thus better tailor patients’ treatments and finally improve their survivals. Although showing promising results, interobserver segmentation variability, feature reproducibility, and model validation are three main challenges of radiomics that need to be addressed in order to translate radiomics studies to clinical applications. These efforts, together with a better knowledge and application of the “Radiomics Quality Score” and Image Biomarker Standardization Initiative reporting guidelines, could improve the quality of sarcoma radiomics studies and facilitate radiomics towards clinical translation.

## 1. Background

Bone and soft-tissue primary malignant tumors or sarcomas represent a large, diverse group of mesenchymal-derived malignancies. They are rare entities with several histological subtypes, and each has an incidence < 1/100,000/year [1,2].

Different bone tumor subtypes have distinct patterns of incidence and among them, osteosarcoma is the most common. Osteosarcoma (OS) and Ewing sarcoma (ES) are more common in the second decade of life, while chondrosarcoma (CS) has a higher incidence in adulthood [1]. Among the over 80 different histological subtypes of soft-tissue sarcomas, liposarcoma, and leiomyosarcoma are the most common. The majority of sarcoma histotypes therefore have an incidence < 2/1,000,000/year. Given the rarity of these tumors, and the complexity of interdisciplinary treatment including radiation therapy, systemic therapy, and surgery, they are managed in tertiary sarcoma centers, able to offer access to the full spectrum of care and expertise [1,2].

Both biopsy and imaging supplement clinical data prior to the beginning of any treatment, with a biopsy representing the reference standard for preoperative diagnosis [1,2]. The biopsy of a suspected primary malignant bone tumor should be performed at the tertiary sarcoma center [3]. An inaccurate biopsy, for instance in the case of sampling errors in large, heterogeneous tumors, may lead to inaccurate diagnosis and thus inadequate treatment. Moreover, there exists the risk of biopsy tract contamination which remains a concern. Imaging plays a pivotal role in diagnosis, staging, response to treatment monitoring, and surveillance for recurrence.

Nowadays, attention is focused on the introduction of artificial intelligence in musculoskeletal (MSK) radiology and the enrollment of radiomic texture analysis as a tool able to non-invasively provide information regarding diagnosis and prognosis [4].

**Figure 1:**
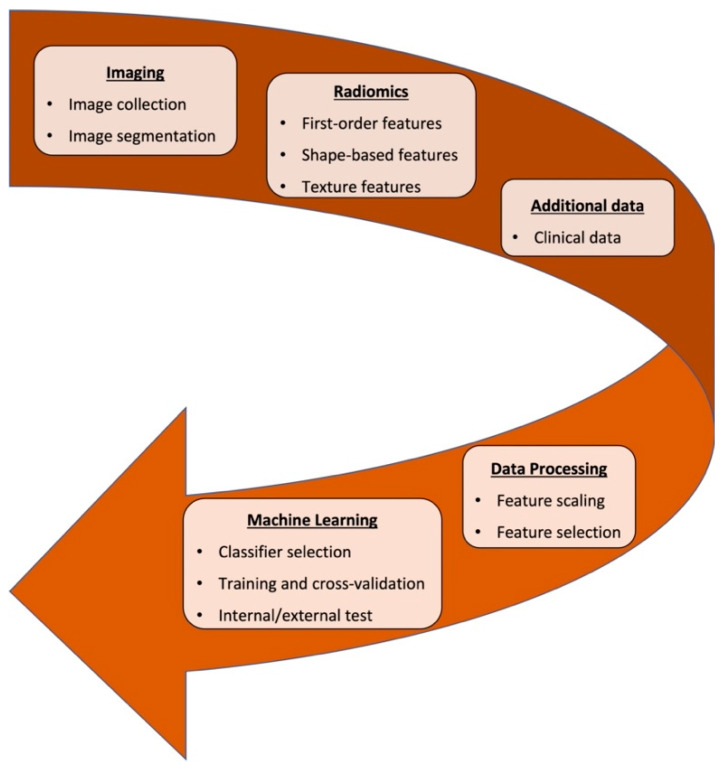
An example of a radiomic workflow. A machine learning classifier can be employed to perform classification tasks based on radiomic features.

The term “radiomics”, from a merging of the terms “radio” and “omics”, indicates the extraction, analysis, and quantitative mapping of many medical image features (i.e., intensity, shape, texture, or wavelet), in relation to clinical prognostic end points and genomics [5]. Radiomic features offer information both on cancer phenotype and the tumor microenvironment which, combined with other pertinent data such as genomics and proteomics and correlated with outcomes data, may provide robust, evidence-based, clinical-decision support systems. To date, several radiomic studies have focused on distinguishing tumor histotype and grading before treatment, monitoring response to therapy and predicting outcome [6]. A schematic diagram illustrating an example of a radiomic workflow, from image collection and segmentation to radiomic feature extraction and selection and, finally, classification model, is shown in Figure 1. Machine learning can be combined with radiomics to perform classification tasks.

In this review, we offer an overview of radiomics achievements in MSK radiology, where the introduction of artificial intelligence and the employment of radiomic texture analysis have resulted in the development of radiomics Magnetic Resonance Imaging (MRI)-based models that could help distinguish different histotypes, low-grade from high-grade sarcomas, predict response to treatment, and overall survival.

## 2. Benign vs. Malignant and Histotype Differentiation

### 2.1. Soft-Tissue Tumors

The accurate diagnosis of soft-tissue lesions with the purpose of discriminating between malignant and benign tumors is crucial for patient management, as their treatment, follow-up, and prognosis differ considerably. The characterization of soft-tissue lesions is still challenging in current clinical practice. MRI is accepted as the standard imaging diagnostic tool for detecting and classifying soft-tissue tumors [7]. Further invasive procedure such as percutaneous biopsy or surgery is often required following MRI in case of uncertain diagnosis. Though biopsy is the usual method of classifying tumor histology before surgery, it may produce complications and sampling, as it is invasive and only evaluates a small sample. Thus, a non-invasive approach to differentiating benign from malignant soft-tissue tumors may avoid an unnecessary invasive procedure and reduce complications.

Radiologists usually utilize certain features related to the biological activity of the tumor to distinguish between benign and malignant soft-tissue tumors, such as size, edema, necrosis, and infiltration of surrounding tissue. These criteria have been demonstrated to have relatively limited performance, with a diagnostic accuracy amounting to 50–85% [7,8,9,10,11,12].

Tumor heterogeneity can be evaluated using histological or imaging data and is a major criterion used to diagnose malignancies [9,13,14,15,16]. Emerging studies show that using computer-assisted diagnostics (CAD) for the quantification of tumor heterogeneity by radiomics-based texture analysis (TA) is a promising tool as it may represent a non-invasive biomarker for differentiation between histological tumor types, grading, response monitoring, and outcome prediction. Radiomics-based TA quantifies the coarseness and regularity of the spatial distribution of pixel grey level values within normal and pathological tissue. Recent studies have demonstrated that macroscopic heterogeneity assessed by medical images likely mirrors underlying histopathological heterogeneity, thus providing a promising diagnostic tool for tumor detection and grading, treatment response, and overall outcome prediction [17,18].

Juntu et al. showed that the accuracy of the T1-weighted (T1WI)-based support vector machine algorithm for discriminating benign vs. malignant soft-tissue tumors was 93%, outperforming radiologists’ classification accuracy [19].

More recently, Wang et al. [20] comprehensively examined the texture features of T1WI and fat-suppressed (FS)–T2 weighted (T2WI) images related to malignancy in soft-tissue tumors. They identified the radiomic features which significantly correlated with malignant soft-tissue lesions, including margin, size, low T2 signal matrix, signal intensity, vessels, myxoid matrix, capsule, and radiomics score, and then constructed a radiomics nomogram by adding the clinical model. The radiomics nomogram (area under the receiver operating characteristic curve (AUC) = 0.96 and 0.88) demonstrated a superior predictive performance to the clinical model based on radiologists’ experience, as well as the radiomics algorithm alone, in two validation sets. In a previous study, there was a demonstrated high accuracy of a radiomics nomogram based on FS-T2WI, outperforming the clinical model for differentiating between benign and malignant soft-tissue masses [21].

Fat-containing soft-tissue tumors are a common clinical entity [22]. Lipomas are the most common soft-tissue tumors and liposarcomas the most common soft-tissue sarcoma [23]. 

In 2020, the World Health Organization committee for Classification of Soft Tissue Tumors [12] distinguished the locally aggressive atypical lipomatous tumor (ALT)/well-differentiated liposarcoma (WDL) from four histologic subtypes of malignant liposarcoma, such as dedifferentiated, myxoid, pleomorphic, and not otherwise specified liposarcoma [12].

Imaging discrimination between lipoma and liposarcoma is a major point, since patient management, treatment, follow-up, and overall outcome differ markedly (5-year survival ranges from almost 100% for lipoma to 60–70% for liposarcoma) [24]. This remains a challenge for conventional MRI examination since a significant number of benign lipomas also have an imaging appearance mimicking ALT/WDL, which may resemble ordinary lipoma. A previous study demonstrated only 69% accuracy for specialized musculoskeletal radiologists in differentiation between these lesions on MRI [25]. 

Thornhill et al. [26] fulfilled the differentiation of lipoma from liposarcoma analyzing texture and shape in multiple sequences (T1WI, T2WI, FS-T2WI, short time inversion recovery (STIR), ce-T1WI) at 1.5 Tesla. They obtained an accuracy of 85%, sensitivity of 96%, and specificity of 91% for textural and morphological features extracted from T1WI sequences compared to radiologists. Given the robustness of T1WI sequences, Malinauskaite et al. [27] relied on this sequence in their study. They showed that radiomics in association with machine-learning methods gave better performances than specialized MSK radiologists in differentiating between lipoma and liposarcoma on preoperative T1WI MRI, obtaining 94.7% diagnostic accuracy, 88.8% sensitivity, and 100% specificity, with positive and negative predicting values of 100% and 78.5%, respectively (AUC = 0.926).

One of the major dilemmas in fat-containing tumors’ diagnosis lies in differentiating lipoma from WDL or ALT. ALT/WDL are locally aggressive and have no potential for metastasis unless dedifferentiation occurs. Even on histological analysis, the diagnosis can be challenging and subjective [28]. To date, the gold standard for the diagnosis of ALT/WDL is fluorescence in situ hybridization (FISH) detection of murine double minute 2 (MDM2) gene amplification [29,30,31]. Several studies evaluated the ability of MRI to differentiate these lesions and suggested size and lipomatous content as reliable imaging discriminators. However, a certain overlap of the imaging features of lipoma and ALT/WDL has been reported, concluding that MRI is unreliable [32,33]. Even post-contrast imaging did not improve the reliability of diagnosis and may occasionally be misleading [34].

A recent study conducted by Pressney et al. [35] demonstrated that radiomics-based texture heterogeneity quantification using fine, medium, and coarse feature scales is able to significantly differentiate between lipoma and ALT/WDL, in particular for medium and coarse texture scales with higher means and lower or negative kurtoses. 

### 2.2. Bone Tumors

Several coarseness factors were also found to be able to discern enchondroma from low-grade chondrosarcoma [36]. Cartilaginous tumors are often an incidental finding in radiological imaging [37]. Discrimination between low-grade chondrosarcoma—renamed atypical cartilaginous tumor in long bones according to the World Health Organization in 2020 [12]—and enchondroma is a challenge both for radiologists and pathologists [38]. 

Lisson and colleagues assessed the diagnostic value of MRI-based 3D texture analysis to estimate intratumor heterogeneity [36] and found several texture parameters with the potential to differentiate between low-grade chondrosarcoma and enchondroma with a high sensitivity, specificity, and accuracy. The most important ones were kurtosis in contrast-enhanced (ce)-T1WI and entropy in T1WI. 

Other studies have looked at the histological differentiation of primary bone tumors. To date, a 3D non-enhanced computed tomography (CT) and an enhanced CT-based radiomics model has been validated as a novel approach to differentiate sacral chordoma and sacral giant cell tumor [39]. Furthermore, a radiomics model based on features extracted from both FS-T2WI and ce-T1WI sequences yielded favorable results and constituted a new technique for the discrimination of OS and ES [40]. Moreover, a multiparametric radiomics signature can accurately differentiate skull base chordoma from chondrosarcoma [41]. Still, radiomics and machine learning have proved useful in spinal lesion differential diagnosis and have demonstrated good diagnostic performances in labeling spinal lesions as either benign or malignant and also in labeling them as benign, primary malignant, or metastases [42].

The main characteristics of the studies discussed in Section 2.1 and Section 2.2 are detailed in Table 1.

## 3. Grading

### 3.1. Soft-Tissue Tumors

Soft-tissue sarcomas (STS) comprise a diverse group of malignant soft-tissue tumors with various prognoses. Among all the prognostic factors, histologic grading into low, medium, or high grade of malignancy is one of the most important in order to predict the probability of distant metastasis and survival [43]. Moreover, treatment plans in STS patients greatly depend on tumor grade. The most important differentiation is between low-grade (G1) and high-grade (G2 or G3), as it directly affects treatment decision in multimodality therapy [44]. 

To date, core needle biopsy is the gold standard for histopathological diagnosis of preoperative STS. It classifies STS into low, medium, or high grade of malignancy on the basis of mitotic counts, differentiation levels, and degrees of necrosis [45,46]. However, it is not always possible to assign a pretherapeutic histologic grade due to insufficient specimens or sampling errors and this will affect the timing of treatment [47].

MRI features have been used by several studies attempting to increase the accuracy of pretherapeutic grade assessment. Both peritumoral features (such as poorly defined margins, peritumoral edema-like signals, and enhancement) and intratumoral heterogeneity have been found to have some potential to predict high-grade tumors [17,48].

To some extent, qualitative features of macroscopic intratumoral heterogeneity such as the presence of intratumoral necrosis, hemorrhage, myxoid degeneration, and calcifications can be assessed by visual observation of routine MR images. However, evaluation of intratumoral heterogeneity at the microscopic level advocates the need for more accurate diagnostic tools.

Radiomics-based texture analysis of MR imaging features allows a deep examination of their distribution in the scanned volume and can be used to assess intratumoral heterogeneity. To date, texture analysis has been successfully applied to imaging studies for the assessment of various neoplasms, aiming to discriminate tumor grades and types before treatment [6,17,49].

A recent small retrospective study conducted by Corino et al. used diffusion weight (DWI) MRI-based radiomics features to distinguish G2 and G3 STS. They reported that the apparent diffusion coefficient (ADC)-based radiomics classifier has the potential to distinguish intermediate from high-grade lesions in STS [50]. However, discriminating G2 from G3 lesions may not provide enough information for multimodality therapy.

Another study demonstrated the role of intratumoral heterogeneity on MRI, assessed by histogram analysis, in discriminating different STS grades [51].They evaluated the role of five histogram parameters (mean, mode, standard deviation (SD), kurtosis, and skewness), automatically extracted from the selected regions of interest (ROI) on T1WI and T2WI images and enhancement ratio (ER) maps. These parameters are members of the first-order statistics of statistical-based texture analysis and reflect the frequency distribution of grey level on images without taking into account spatial factors [17,49]. The study demonstrated that intratumoral heterogeneity evaluated by quantitative features on MR images, in particular skewness and kurtosis, has the potential to predict the differentiation of different histologic grades of STS. 

Zhang et al. also developed a non-invasive radiomics tool to determine the histopathological grades of soft-tissue tumors and thus predict biologic behavior [52]. They automatically extracted first-order statistics, shape- and size-based features, texture features, and higher-order statistical features from FS-T2WI MR images and compared the performance of three different radiomics classifiers. They showed that when training with the support vector machine classification method (SVM), the radiomics classifier had better performances than a biopsy in discriminating STS histopathological grades [52].

Peeken et al. assessed the predictive performances of radiomic models based on different MRI sequences (T2FS, T1FSGd, and a combined model). The three models achieved an area under the receiver operator characteristic curve (AUC) of 0.78, 0.69, and 0.76, respectively [53]. 

### 3.2. Bone Tumors

As seen for STS, the clinical outcome of cartilaginous tumor mostly depends on the histological grading, as the 10-year overall survival ranges from 88% for low-grade/atypical cartilaginous tumors to 62% and 26% for grade 2 and grade 3 chondrosarcoma, respectively [54]. Moreover, treatment drastically changes among different histopathological grades of cartilaginous tumors [1]. However, the downgrading of chondrosarcoma may derive from sampling errors in preoperative biopsy [55]. Moreover, a significant interobserver variability in tumor grading has been observed, even among specialized bone pathologists. Thus, integrating imaging data to clinical data and biopsy is of pivotal importance and MRI is the gold standard [56]. Useful imaging characteristics in chondrosarcoma grading have been found to be bone expansion, periosteal reaction, soft-tissue mass, and tumor length, yielding a diagnostic accuracy > 90% [57], as well as bone marrow edema, cortical thickening, and destruction and soft-tissue edema [57], while you cannot differentiate different chondrosarcoma grades using diffusion-weighted-(DWI) MRI [58].

Fritz et al. evaluated the diagnostic accuracy of morphologic MRI and MRI-based bidimensional texture analysis for chondrosarcoma grading in a series of 53 chondromas and 63 low-to-high-grade cartilaginous tumors [59]. They obtained the highest diagnostic performances for differentiation of benign from malignant, as well as benign from low-grade tumor, with a combination of both morphologic MRI and texture analysis predictors, but were unable to differentiate low-grade from high-grade lesions [59]. Data mining and machine learning could address this limitation of classical statistical approaches [60]. 

Recent studies aimed to evaluate the diagnostic accuracy of machine learning for tumor grading of cartilaginous bone tumors based on radiomic parameters extracted from MRI. Gitto and colleagues attempted to discriminate low-grade/atypical cartilaginous tumors from higher-grade lesions extracting radiomic data from unenhanced T1WI and T2WI sequences of MRI [61]. They combined texture analysis with machine learning, and performed automatic feature selection through a Random Forest wrapper whose output comprised four features derived from T1WI sequences. Afterwards, the performance of a locally weighted ensemble classifier was evaluated on the test cohort, and was showed to be as good as an experienced musculoskeletal radiologist (AUC = 0.78). In agreement with these results, more recently they attempted to develop a machine-learning classifier based on preoperative CT radiomic features to discriminate between atypical cartilaginous tumors and high-grade chondrosarcomas of long bones [62]. The CT radiomics-based machine-learning classifier achieved 75% accuracy overall, 81% accuracy in identifying atypical cartilaginous tumors, and 70% accuracy in identifying higher-grade chondrosarcomas, and still there was no difference in comparison with an experienced radiologist (*p* = 0.75). Additionally, Gitto et al. recently obtained 92% accuracy in differentiating atypical cartilaginous tumor from grade 2 chondrosarcoma of long bones using T1WI MRI radiomics-based machine learning, with no difference compared to an experienced musculoskeletal oncology radiologist (*p* = 0.134) [63].

Altogether, these results suggest that even though qualitative image assessment still plays a central role in the diagnosis and tumor grade discrimination, a radiomics-based machine learning classification model of low-to-high-grade tumors is a promising tool for preoperative tumor characterization.

The main characteristics of the studies discussed in Section 3.1 and Section 3.2 are detailed in Table 2.

## 4. Treatment Response

### 4.1. Soft-Tissue Tumors

The standard of care for patients with locally advanced high-grade STS is being reconsidered since anthracycline-based neoadjuvant chemotherapy (NAC) has been shown to improve overall and metastasis-free survivals of patients [64,65,66]. The prediction of response to NAC is of pivotal importance since any difficulty with the prediction may hamper personalized medicine strategies that depend on pathological examination results.

In a recent study, the accuracy of Choi criteria to predict a very good pathological response (defined as <10% viable cells on surgical specimens) was demonstrated to be 74.1% [67,68]. In another study, a decrease in contrast enhancement of –30.5% between two MRIs with optimized acquisition timing after contrast-agent injection yielded an accuracy of 82.8% [69]. In a prospective study of 50 patients, a decrease of >35% of maximum standardized uptake value (SUVmax) at an early evaluation with ^18^FDG-PET-CT provided an AUC of 0.83 [70,71]. Despite these encouraging results, response evaluation is still based on response evaluation criteria in solid tumors (RECIST). However, shortening of the longest diameter is not an adequate criterion to predict therapeutic response to NAC of STS, since they usually do not shrink. MRI evaluation of STS during NAC can point out a wide range of morphologic changes conjugating fibrotic and necrotic processes, infarction, bleeding, redifferentiation, or selection of resistant components. These lead to a change in tumor heterogeneity that could be quantified with shape and texture features making STS particularly suitable to the radiomics approach for the evaluation of tumor response to treatment. The traditional radiomics system analyzes features extracted from single-phase medical images, thus neglecting the changes that occurred during treatment or follow-up. Delta-radiomics quantifies the change in radiomic features during or after treatment, and is therefore more appropriate for the evaluation of tumor response to treatment and provides a potential tool for precision medicine [72]. The delta-radiomics approach has been demonstrated to be predictive of prognoses and metastases’ occurrence in previous studies conducted on non-small cell lung cancer [73,74].

A retrospective study by Crombé et al. investigated the potential of an MRI T2-based delta-radiomics approach to improve early response assessment in high-grade STS patients treated by anthracycline-based NAC [75]. A threshold of <10% viable cells on surgical specimens defined good histological response (good-HR). Three senior radiologists reported RECIST response status and performed a semantic analysis of the MRI at baseline and early evaluation after NAC, reporting changes in tumor volume compatible with fibrosis and/or necrosis, margin definition, surrounding edema, and peritumoral enhancement. After 3D manual segmentation of tumors at baseline and early treatment stages, absolute changes in 33 first- and second-order texture and shape features were calculated. An association with response was observed neither for RECIST 1.1 (*p* = 0.112) nor for semantic radiological variables (range of *p*-values: 0.134–0.490), with the exception of an edema decrease (*p* = 0.003). Whereas 14 shape and texture features were associated with treatment response (range of *p*-values: 0.002–0.037), the highest diagnostic performance on the training cohort was obtained with three features: Δ_Histogram_Entropy, Δ_Elongation, Δ_Surrounding_Edema, (AUC = 0.86, accuracy = 88.1%, sensitivity = 94.1%, and specificity = 66.3%). On the test cohort, this model provided an accuracy of 74.6%. These preliminary results indicate that a T2-based delta-radiomics approach might be able to ameliorate performances in early response assessment in STS patients.

A more recent longitudinal imaging study explored radiomics features from longitudinal DWI MRI for the assessment of treatment response in patients with localized STS undergoing hypofractionated preoperative radiotherapy (RT) [76]. A support vector machine (SVM) model built to predict the treatment effect score was used both with mean ADC or delta ADC and with radiomics features extracted from longitudinal DWI and tumor ADC maps acquired at three time points. The prediction performance of mean ADC or delta ADC alone was poor (AUC < 0.74), whereas including delta radiomics of mid- or post-treatment relative to the baseline substantially improve the prediction.

### 4.2. Bone Tumors

In addition, for high-grade osteosarcoma (HOS), the gold standard for treatment is NAC, followed by surgical resection and adjuvant chemotherapy [77]. The long-term survival rate of localized osteosarcoma patients has improved markedly after the introduction of NAC, with a 5-year survival rate of approximately 60–70% [78]. However, in patients with poor histologic responses after NAC, their prognoses are still poor [78,79]. Thus, the accurate prediction of histologic responses to NAC in patients with HOS is a critical point for treatment planning and prognoses [80]. 

Changes in tumor volume have to date been proposed as a prediction factor to treatment response. However, osteosarcoma does not significantly shrink after NAC [81], but the tumor may undergo necrosis or change in vascularization or become cystic, with no significant change in tumor size.

Several prediction models have been developed to distinguish good responders from others with HOS, based on ^18^FGD PET/CT or MRI [81,82,83,84]. Most models have focused on qualitative description of medical images or used a mean value to represent whole tumors, which may neglect tumor heterogeneity and have limitations in predicting therapeutic responses.

A recent retrospective study developed and validated a delta-radiomics nomogram to evaluate pathologic responses after NAC in patients with HOS [85]. This work aims to identify the poor response HOS patients by combining pre- and post-treatment CT data. There were 7 intensity features and 53 texture features extracted from each region of interest (ROI) on the CT images before and after NAC, with radiomics signatures built for comparison purposes as well. A radiomics nomogram was then developed by combining the delta-radiomics signature with independent clinical factors such as the occurrence of new pulmonary metastases. The study showed that the delta-radiomics signature performed better than single-CT-based radiomics signatures in both training and validation cohorts in discriminating between the pathologic good response (necrosis fraction ≥ 90%) group and the non-pathologic good response (necrosis fraction < 90%) group (*p* < 0.0001). As well, the delta-radiomics nomogram showed good discrimination ability with AUC 0.871 and 0.843 in the training and validation cohorts, respectively, suggesting it could be used to better tailor proper chemotherapy and treatment plans.

The main characteristics of the studies discussed in Section 4.1 and Section 4.2 are detailed in Table 3.

## 5. Local Recurrence and Metastasis 

### 5.1. Soft-Tissue Tumors

The incidence of local recurrence (LR) for STS is about 6.5% and 25% and is related to poor prognosis [86]. The American College of Radiology (ACR) Appropriateness Criteria guidelines suggest MRI as the most proper imaging examination for LR surveillance of musculoskeletal STS [87]. However, despite MRI being capable of differentiating local recurrence from post-surgical changes (i.e., edema, hematoma, inflammation, and scarring), they can sometimes mimic local recurrence on T1WI, T2WI, and post-contrast sequences [87,88,89,90]. Postoperative inflammation and fibrosis may share many characteristics with tumors on a conventional MRI and can occasionally appear mass-like [91]. Moreover, some LR are not nodules but plaque-like “tails” of tumor on MRI, or they may be of a low signal intensity on T2WI images with only architectural distortion visible on a T1WI study due to the tumoral presence. In these cases, radiomics may be a valid aid to radiologists in the detection of LR [90].

A recent prospective study on a small number of patients hypothesizes that MRI radiomics analysis of patients undergoing follow-up for STS allows the differentiation of LR from normal tissue [92]. They showed that radiomics features extracted from T1WI MRI images, FS-T2WI MRI images, and T1WI post-gadolinium (Gd) sequences can differentiate LR from normal tissue better than conventional MRI (AUC = 0.96 for radiomics based on T1WI post-Gd).

About 25% of all patients with STS develop distant metastases [93]. In those with high-grade tumors, the metastatic recurrence rate increases to approximately 50% [94]. The main site of distant metastases in patients with STS of the extremities are the lungs (80% of metastatic cases) [95]. The development of lung metastases affects both prognosis and management of STS patients, thus making the prediction of lung metastases risk of great interest in the course of STS management. As discussed above, radiomics tools for the study of tumor heterogeneity yield valuable information about tumor aggressiveness. 

Vallières and colleagues developed a joint model merging FDG-PET and MRI-extracted texture features for an early assessment of the risk of lung metastases in STS patients [96]. Nine non-texture features (shape features and SUV metrics) and forty-one texture features were extracted from the ROI of single FDG-PET, T1WI, and FS-T2WI scans and fused FDG-PET/T1WI and FDG-PET/FS-T2WI scans. In agreement with other studies [97,98], SUVmax was significantly related to lung metastasis risk in STS patients. A significant positive association was also shown to exist with Percent Inactive, being the volume of inactive FDG-PET regions of tumors and lung metastases’ occurrence. However, texture analysis better characterizes intratumoral heterogeneity and better predicts lung metastases risk. In particular, merging MRI information with that of FDG-PET performs better than MRI or FDG-PET alone (AUC of four texture features extracted from FDG-PET/T1WI and FDG-PET/FS-T2WI scan = 0.984 ± 0.002) [96]. 

### 5.2. Bone Tumors

The incidence of local or distant relapse in patients with localized osteosarcoma is about 30–40%, and it results in a decrease of the 5-year survival rate to 23%–29% [99]. The majority of these recurrence occurs in the first year of treatment (early relapse) [100]. Accurate prediction of early relapse in osteosarcoma is still a challenge that could take advantage of radiomics-based evaluation of tumor heterogeneity on MRI.

Chen et al. developed and validated an MRI-based radiomics nomogram from retrospective multicenter datasets to predict the risk of early relapse (≤1 year) in osteosarcoma after surgery [101]. Radiomics features were extracted from contrast-enhanced (ce)-T1WI images and features were extracted through a LASSO regression system. A radiomics nomogram was constructed by incorporating MRI-assessed predictors such as joint invasion and perivascular involvement. It was shown to be capable of predicting early relapses of osteosarcoma, providing a potential tool to improve personalized therapy.

The main characteristics of the studies discussed in Section 5.1 and Section 5.2 are detailed in Table 4.

## 6. Overall Survival

### 6.1. Soft-Tissue Tumors

STS behavior largely differs, ranging from indolent tumors to highly aggressive disease. Besides tumor size, the French Federation of Cancer Center’s histologic grading system is the other major prognostic factor for STS. It takes into consideration tumor differentiation, mitotic activity, and necrosis [102]. Several studies have corroborated its prognostic value in terms of local recurrence-free survival, metastasis-free survival, and overall survival [43,103,104]. However, tumor heterogeneity and the possible underestimation of histologic grade because of sampling errors remain a concern. As discussed above, biopsy grade can be corrected using imaging findings, so it has been hypothesized that imaging features associated with grade would correlate with patients’ prognoses.

Crombé and colleagues conducted a retrospective single-center study with the aim of assessing the relationship between conventional MRI features and high tumor grade and to determine consequent information regarding patient outcomes [105]. They evaluated qualitative characteristics of images at T2WI, T1WI precontrast, and T1WI postcontrast MRI. Kaplan–Meier curves and multivariable Cox models were used to evaluate possible associations of these features with overall survival and metastasis-free survival. Based on multivariable analysis, there were three independent MRI features (presence of necrosis, heterogeneous signal intensities at T2W, and peritumoral enhancement) found to be associated with grade 3 STS. No metastatic relapses or deaths were reported in the absence of these three relevant MRI features. These findings hint that baseline MRI studies may be complementary to histologic grade in providing prognostic information about STS patients.

Prior studies reported promising results for quantitative imaging biomarkers of STS as prognostic factors. These mainly focused on positron emission tomography/computed tomography (PET/CT) and found associations of quantitative features and structural features (e.g., tumor boundary heterogeneity) with patient outcomes [97,98,106]. More recently, radiomics has emerged as a promising tool capable of capturing complex image characteristics and providing a quantitative analysis of texture heterogeneity. 

Radiomics features extracted from STS have been found to be possibly associated with the risk of developing distant metastases and overall survival in STS. Spraker et al. [107] evaluated the hypothesis that quantitative imaging features extracted from pretherapy T1WI MR images would be predictive of overall survival in patients with STS. They extracted 30 radiomic features from pretreatment T1WI ce-MRI of two independent cohorts of patients with stage 2–3 STS. After feature selection, they trained three models for predicting overall survival: a clinical-only model (C) containing only age and grade as predictors, a radiomics-only model (R), and a combined model (C + R). There were two main findings. First, radiomic features alone were together significantly predictive of overall survival. Second, the combined model (C + R) outperformed the predictive performances of overall survival compared with clinical features alone.

These results were similar to those obtained by Peeken et al. After evaluating the performance of their MRI-based radiomics models in discriminating between low-grade (G1) and high-grade (G2/G3) STS, they went further by analyzing the usefulness of radiomics models for prognostic assessment [53]. Radiomics models directly trained to predict overall survival only showed moderate predictive performances. However, radiomics nomograms were created by combining the American Joint Committee on Cancer (AJCC) staging system (7th edition) with the radiomics grading models, for prognostic assessment. Combining the FS-T2WI radiomics model into a nomogram with AJCC clinical staging showed the best predictive performance for overall survival, above clinical staging alone.

They also conducted a similar study on pre-radiotherapy-planning CT scans and investigated whether quantitative imaging features extracted from CT scans performed for radiotherapy planning provided prognostic information [108]. After features extraction and reduction, machine learning modeling for the prediction of grading, overall survival, and distant (DPFS) and local (LPFS) progression-free survival were fulfilled followed by external validation. They evaluated a radiomics model, a clinical model, and two combined models: one obtained by combining clinical features and the tumor volume and the other trained on all radiomics and clinical features. They observed that radiomics models were able to differentiate grade 3 from non-grade 3 STS (AUC: 0.64). Moreover, radiomic models showed better predictive performances for patients’ overall survival, DPFS, and LPFS, compared to a clinical model. Still, the combined model achieved the best performance for overall survival.

### 6.2. Bone Tumors

As is the case with STS, osteosarcomas also differ widely for aggressiveness and tumor behavior. Therefore, the identification of prognostic biomarkers is crucial for osteosarcoma treatment planning, especially in patients with localized osteosarcoma. Radiomics aims to quantify heterogeneous aspects of tumor images with the admission that this information is associated with tumor biology and behavior [109].

A recent study validated the hypothesis that a radiomic signature extracted from DWI-MRI can outperform predictive performances compared with clinical factors alone in localized osteosarcoma. Multivariate Cox regression was used to validate the radiomics signature as an independent biomarker showing that the radiomics signature was predictive of overall survival. The combined model incorporating radiomics and clinical factors still resulted in better performances in terms of survival (C-index: 0.813; 95% CI: 0.75, 0.89) when compared both with radiomics (C-index: 0.712; 95% CI: 0.65, 0.78) and clinical models alone [109]. 

In another study, radiomics features were extracted from the pretreatment diagnostic computed tomography images of patients with HOS [110], a clinical model was constructed by using clinical factors only (stage and tumor volume), and a radiomics nomogram was developed by incorporating the radiomics score and clinical factors. The radiomics nomogram showed better performance than the clinical model, both in terms of better calibration and classification capacity (AUC 0.86 vs. 0.79 for the training cohort, and 0.84 vs. 0.73 for the validation cohort) and prediction of survival and non-survival group.

The main characteristics of the studies discussed in Section 6.1 and Section 6.2 are detailed in Table 5.

## 7. Limitations and Conclusions

Radiomics has now become one of the main fields of research in oncologic imaging. It arises from the underlying hypotheses that quantitative imaging features reflect the molecular phenotype of tumor and that predictive models can be developed and improved by integrating radiomics data with non-radiological and “-omics”. Therefore, radiomics appears complementary to histopathological and molecular analyses to predict tumor grading, identify relevant subgroups of patients, predict response to multimodality therapy, and thus better design personalized treatments to finally improve patients’ survival.

However, no oncologic radiomics studies have yet translated to clinical applications. Hence, to overcome the turning point between proofs of concept and real-life application, one of the major issues to be addressed is that of radiomic feature reproducibility and model validation which vary widely among the studies dealing with musculoskeletal sarcomas [111,112,113]. In particular, a certain degree of interobserver segmentation variability highlights the need for a preliminary reproducibility analysis in radiomic studies [114]. Machine learning can be combined with radiomics to perform model validation [115,116].

Although showing promising results, improvements in study design, validation, and open science are needed to make sarcoma radiomics studies reproducible with an acceptable level of evidence needed. These efforts, together with a better knowledge and application of the “Radiomics Quality Score” and Image Biomarker Standardization Initiative [112] reporting guidelines, could improve the quality of sarcoma radiomics studies and facilitate radiomics towards clinical translation. 

## Figures and Tables

**Table 1:** Predictive performances of radiomics models in discriminating benign vs. malignant tumors.

Authors	Year	Type of Tumor	Technique	Sequences	Predictive Performances	Radiomics Nomogram (Radiomics Combined with Clinical Features)
Juntu et al. [19]	2010	Soft-tissue tumors	MRI	T1WI	AUC = 0.91	N/A
Wang et al. [20]	2020	Soft-tissue tumors	MRI	T1WI, FS-T2WI	AUC = 0.86, 0.82	AUC = 0.96, 0.88
Malinauskaite et al. [27]	2020	Lipoma vs. liposarcoma	MRI	T1WI	AUC = 0.926	N/A
Pressney et al. [35]	2020	Lipoma vs. ALT/WDL	MRI	PDWI	AUC = 0.8	N/A
Lisson et al. [36]	2018	Enchondroma vs. chondrosarcoma G1	MRI	T1WI (ce)-T1WI	AUC = 0.851, 0.822 AUC = 0.876, 0.826	N/A

**Table 2:** Predictive performances of radiomics models in grading tumors.

Authors	Year	Type of Tumor	Technique	Sequences	Predictive Performances
Corino et al. [50]	2018	STS (G2 vs. G3)	MRI	ADC	AUC = 0.85, 0.87
Xiang et al. [51]	2019	STS (G1 vs. G2 vs. G3)	MRI	ER (Enhancement Ratio) maps	AUC = 0.747, 0.684
Zhang et al. [52]	2019	STS (G1 vs. G2 vs. G3)	MRI	FS-T2WI	AUC = 0.92 (SVM)
Peeken et al. [53]	2019	STS (G1 vs. G2 vs. G3)	MRI	T2WI ce-T1WI combined	AUC = 0.78 AUC = 0.69 AUC = 0.76
Fritz et al. [59]	2018	Chondrosarcomas (G1 vs. G2 vs. G3)	MRI	T1WI, ce-T1WI	Not significant
Gitto et al. [61]	2020	Atypical cartilaginous tumor vs. G2-G4 chondrosarcoma	MRI	T1WI T2WI	AUC = 0.78
Gitto et al. [62]	2021	Atypical cartilaginous tumor vs. G2-G4 chondrosarcoma	CT	CT	AUC = 0.78
Gitto et al. [63]	2022	Atypical cartilaginous tumor vs. G2 chondrosarcoma	MRI	T1WI	AUC = 0.94

**Table 3:** Performances of radiomics models in response to treatment prediction.

Authors	Year	Type of Tumor	Treatment	Technique	Sequences	Δ_Radiomics Predictive Performances	Δ_Radiomics Nomogram Predictive Performances
Crombé et al. [75]	2019	G3 STS	NAC	MRI	T2WI	AUC = 0.86	N/A
Gao et al. [76]	2020	G3 STS	RT	MRI	ADC	AUC = 0.85	N/A
Lin et al. [85]	2020	HOS	NAC	CT	N/A	AUC = 0.868, 0.823	AUC = 0.871, 0.843

**Table 4:** Performances of radiomics models in the prediction of local recurrence and metastasis.

Authors	Year	Type of Tumor	Prediction/ Discrimination	Technique	Sequences	Radiomics Model Performances	Radiomics + Clinical Features Performances
Tagliafico et al. [92]	2019	STS	Fibrosis vs. LR	MRI	ce-T1WI	AUC = 0.96	N/A
Vallières et al. [96]	2015	STS	Lung metastasis risk	FDG-PET MRI	FDG-PET/T1WI, FDG-PET/FS-T2WI	AUC = 0.984	N/A
Chen et al. [101]	2020	HOS	LR	MRI	ce-T1WI	AUC = 0.887, 0.763	AUC = 0.907, 0.811

**Table 5:** Performances of radiomics models in the prediction of overall survival, distant progression-free survival, and local progression-free survival.

Authors	Year	Type of Tumor	Prediction/ Discrimination	Technique	Sequences	Radiomics Model Performances	Radiomics + Clinical Features Performances
Spraker et al. [107]	2019	STS	OS	MRI	ce-T1WI	C-index = 0.68	C-index = 0.78
Peeken et al. [53]	2019	STS	OS	MRI	FS-T2WI ce-T1WI combined tumor volume	C-index = 0.55 C-index = 0.60 C-index = 0.60 C-index = 0.54	C-index = 0.67 C-index = 0.70 C-index = 0.66 C-index = 0.71
Peeken et al. [108]	2019	STS	OS DPFS LPFS	CT	N/A	C-index = 0.73 C-index = 0.68 C-index = 0.77	C-index = 0.76
Zhao et al. [109]	2019	HOS	OS	MRI	DWI	C-index = 0.712	C-index = 0.813
Wu et al. [110]	2018	HOS	OS	CT	N/A	AUC = 0.79, 0.73	AUC = 0.86, 0.84
